# Physicians' Visiting List for 1882

**Published:** 1881-12

**Authors:** 


*The Physicians' Visiting List for* 1882.—Publishers: Lindsay & Blakiston, Philadelphia.

We are as usual in receipt of this welcome and useful Visiting List, which we have used for a number of years as a dental appointment book. The present edition contains almanac, table of signs, ready method (Hall's) in asphyxia, poisons and anti dotes, metre or French decimal system of weights and measures, posological table, table for calculating period of utero-gestation, blank leaves, etc., rendering it a convenient and well arranged publication for the physician and dentist.

